# IL-6 and IL-8 Serum Levels Predict Tumor Response and Overall Survival after TACE for Primary and Secondary Hepatic Malignancies

**DOI:** 10.3390/ijms19061766

**Published:** 2018-06-14

**Authors:** Sven H. Loosen, Maximilian Schulze-Hagen, Catherine Leyh, Fabian Benz, Mihael Vucur, Christiane Kuhl, Christian Trautwein, Frank Tacke, Philipp Bruners, Christoph Roderburg, Tom Luedde

**Affiliations:** 1 Department of Medicine III, University Hospital RWTH Aachen, Pauwelsstraße 30, 52074 Aachen, Germany; sloosen@ukaachen.de (S.H.L.); ctrautwein@ukaachen.de (C.T.); ftacke@ukaachen.de (F.T.); croderburg@ukaachen.de (C.R.); 2 Department of Diagnostic and Interventional Radiology, University Hospital RWTH Aachen, Pauwelsstraße 30, 52074 Aachen, Germany; mschulze@ukaachen.de (M.S.-H.); ckuhl@ukaachen.de (C.K.); pbruners@ukaachen.de (P.B.); 3 Division of Gastroenterology, Hepatology and Hepatobiliary Oncology, University Hospital RWTH Aachen, Pauwelsstraße 30, 52074 Aachen, Germany; catherine.leyh@rwth-aachen.de (C.L.); fabian.benz@med.uni-heidelberg.de (F.B.); mvucur@ukaachen.de (M.V.)

**Keywords:** cancer, TACE, cytokines, HCC

## Abstract

While surgical resection represents the standard potentially curative therapy for liver cancer, transarterial chemoembolization (TACE) has evolved as a standard therapy for intermediate-stage hepatocellular carcinoma (HCC) as well as liver metastases. However, it is still not fully understood which patients particularly benefit from TACE. Cytokines represent a broad category of signaling molecules that might reflect concomitant inflammation as an adverse prognostic factor. Here, we evaluated the role of interleukin (IL)-6, IL-8, and CC-chemokine ligand (CCL)22 as biomarkers in the context of TACE treatment. Cytokine serum levels were analyzed by multiplex immunoassay in 54 patients (HCC: *n* = 44, liver metastases: *n* = 10) undergoing TACE as well as 51 healthy controls. Patients with primary and secondary liver cancer showed significantly elevated levels of IL-6 and IL-8 but not CCL22 compared to healthy controls. Interestingly, low pre-interventional levels of IL-6 and IL-8 were predictors for an objective response after TACE in binary logistic regression. In contrast, patients with high pre-interventional IL-6 and IL-8 serum levels not only poorly responded to TACE but had a significantly impaired overall survival. Serum levels of IL-6 and IL-8 represent promising biomarkers for patients undergoing TACE and might help to pre-interventionally identify patients who particularly benefit from TACE regarding objective treatment response and overall survival.

## 1. Introduction

Hepatocellular carcinoma (HCC), the most common primary liver malignancy, shows an increasing incidence and is associated with an unfavorable prognosis [1]. Liver metastases frequently arise in the clinical course of various gastrointestinal malignancies such as colorectal cancer (CRC) and represent a limiting factor for long-term survival [2,3]. For both primary and secondary liver tumors, surgical resection represents the standard potentially curative treatment option in daily practice but is often not feasible due to advanced tumor stage or limited liver function at time of diagnosis [3]. Transarterial chemoembolization (TACE) has evolved as a standard therapeutic option for patients with intermediate-stage, irresectable HCC (Barcelona Clinic Liver Cancer (BCLC) stage B), and several studies have confirmed the general therapeutic benefit of TACE for these patients [4]. While systemic chemotherapy represents the standard of care for irresectable liver metastases, e.g., from CRC, TACE is increasingly applied in patients with liver-dominant colorectal metastases after failure of surgery or systemic chemotherapy [5]. However, response rates to TACE are heterogeneous and it is not fully understood which patients benefit most from TACE therapy in terms of tumor response and overall survival [4,6]. As multimodal therapeutic approaches for both primary and secondary liver cancer are gaining complexity in terms of treatment timing as well as potential treatment alternatives (e.g., selective internal radiation therapy (SIRT) or radiofrequency ablation (RFA)), pre-interventional stratification algorithms are of increasing importance to identify patients who represent particularly good candidates for TACE therapy. Several predictive algorithms such as the ART or SNACOR score have been suggested but are mainly based on imaging techniques and the patients’ liver function while aspects of the individual tumor biology only play a minor role [7,8].

Cytokines could potentially reflect distinct inflammatory mechanisms during tumor progression and might therefore serve as prognostic biomarkers in cancer patients [9,10]. As such, CCL22 has been described as a pro-malignant cytokine, and high serum levels of CCL22 were associated with advanced stage of disease as well as an impaired prognosis in patients with gastric cancer and breast cancer [11,12]. Interleukin (IL)-8 and IL-6 both play an important role in various cancer signaling pathways [13,14]. Elevated circulating levels of IL-6 and IL-8 are associated with a reduced overall survival in patients with lung cancer and pancreatic adenocarcinoma [15,16]. However, only little is known about circulating cytokine levels in the context of TACE treatment.

Here, we aimed at evaluating circulating levels of IL-6, IL-8, and CCL22 as serum markers for patients undergoing TACE for primary and secondary liver cancer.

## 2. Results

### 2.1. Serum Levels of IL-8 and IL-6 Are Elevated in Patients with Primary or Secondary Liver Cancer

**Figure 1:**
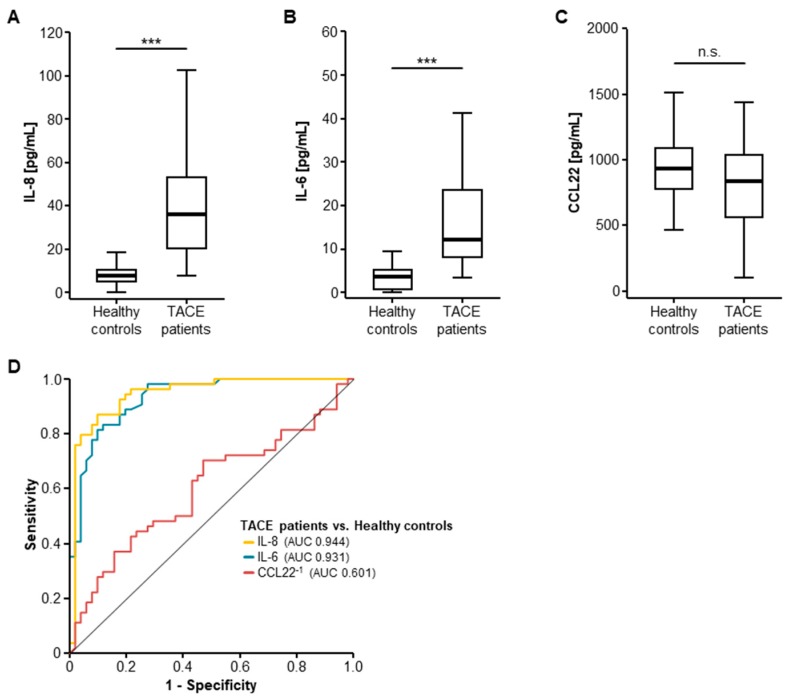
Serum levels of IL-6 and IL-8 but not CCL22 are elevated in patients with hepatic malignancies. (**A**,**B**) Pre-interventional serum concentrations of IL-8 and IL-6 are significantly elevated in patients with primary and secondary liver cancer; (**C**) Serum levels of CCL22 are unaltered between these patients and healthy controls; (**D**) ROC curve analysis shows AUCs of 0.944 and 0.931 for IL-8 and IL-6 regarding the discrimination between TACE patients and healthy controls. (* *p* < 0.05; ** *p* < 0.01; *** *p* < 0.001; n.s.: *p* > 0.05).

We first compared the baseline serum level of IL-8, IL-6, and CCL22 in patients who underwent TACE for primary and secondary liver tumors as well as healthy controls. Here, pre-interventional serum concentrations of IL-8 and IL-6 were significantly elevated in patients with liver cancer compared to healthy controls (Figure 1A,B, Table 1), while serum levels of CCL22 were unaltered between these groups (Figure 1C). In line with this, receiver operating characteristic (ROC) curve analyses revealed area under the curve (AUC) values of 0.944 and 0.931 for IL-8 and IL-6 for the discrimination between liver cancer patients and healthy controls (Figure 1D).

### 2.2. Pre-Interventional Serum Levels of IL-8 and IL-6 Predict an Objective Response to TACE Therapy

We next investigated if pre-interventional cytokine serum levels have a predictive value regarding the individual response to TACE therapy. We therefore divided our cohort into patients with an objective response (OR; including patients with a complete or partial remission) and patients who showed a non-objective response (non-OR; including patients with stable or progressive disease) after TACE [17].

**Figure 2:**
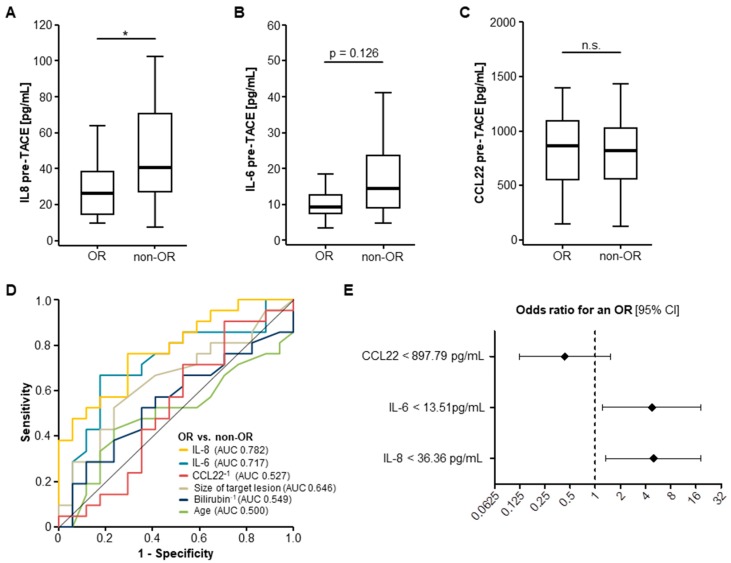
Pre-interventional serum levels of IL-8 and IL-6 predict an objective response after TACE. (**A**) Pre-treatment serum levels of IL-8 are significantly lower in patients who showed an objective response (OR) to TACE compared to non-responding (non-OR) patients; (**B**) Initial serum IL-6 levels similarly show a strong trend towards lower serum concentrations in OR patients; (**C**) Circulating levels of CCL22 are unaltered between OR and non-OR patients; (**D**) ROC curve analysis for the discrimination between OR and non-OR patients; (**E**) Univariate binary logistic regression analysis reveals circulating IL-6 and IL-8 but not CCL22 levels as predictors of an objective response to TACE (black diamond: odds ratio, error bars: 95% confidence interval (CI). (* *p* < 0.05; ** *p* < 0.01; *** *p* < 0.001; n.s.: *p* > 0.05).

In this analysis, pretreatment levels of IL-8 were significantly lower in patients who showed an OR after TACE compared to non-responding (non-OR) patients (Figure 2A). Median pre-interventional IL-6 levels were also lower in OR patients, though statistical significance was not reached (*p* = 0.126, Figure 2B), while circulating levels of CCL22 before TACE were unaltered between OR and non-OR patients (Figure 2C). In line with this, ROC curve analysis for the discrimination between OR and non-OR patients revealed AUC values of 0.782 and 0.717 for IL-8 and IL-6 serum levels, which were superior to bilirubin serum levels as a surrogate for liver function (AUC: 0.549), size of target lesion (AUC: 0.646), and the patients’ age (AUC: 0.500, Figure 2D). On the contrary, circulating levels of CCL22 could not discriminate between OR and non-OR patients (AUC: 0.527, Figure 2D).

To further substantiate a potential predictive value of IL-8 and IL-6 for discrimination between OR and non-OR patients, we next performed univariate binary logistic regression analysis. Importantly, in this analysis, pre-interventional serum levels of IL-8 and IL-6 below our defined ideal cut-off values (IL-8: 36.36 pg/mL, IL-6: 13.51 pg/mL) were significant predictors for an OR following TACE treatment. The odds ratios of IL-8 and IL-6 serum levels for the prediction of an OR in this setting were 4.978 (*p* = 0.016; 95% CI: 1.346–18.403) and 4.773 (*p* = 0.024; 95% CI: 1.229–18.530), respectively (Figure 2E). Of note, serum levels of CCL22 had no predictive value for an OR (odds ratio: 0.432, *p* = 0.190, 95% CI: 0.123–1.515, Figure 2E).

We finally evaluated if pretreatment serum cytokine levels directly correlated with a change of size of the target lesion after TACE. Interestingly, serum levels of IL-8 before TACE treatment significantly correlated with the target lesions’ longitudinal change of size after TACE, meaning that target lesions of patients with high IL-8 levels were more likely to progress after TACE (Supplementary Figure S1A). Pre-TACE IL-6 serum concentrations showed a similar trend, though statistical significance was not reached (*p* = 0.071, Supplementary Figure S2B). In contrast, serum CCL22 levels did not correlate with a change of size of the target lesion (Supplementary Figure S1C).

### 2.3. Elevated Pre-Interventional Serum Levels of IL-8 and IL-6 Are Associated with a Reduced Overall Survival after TACE

**Figure 3:**
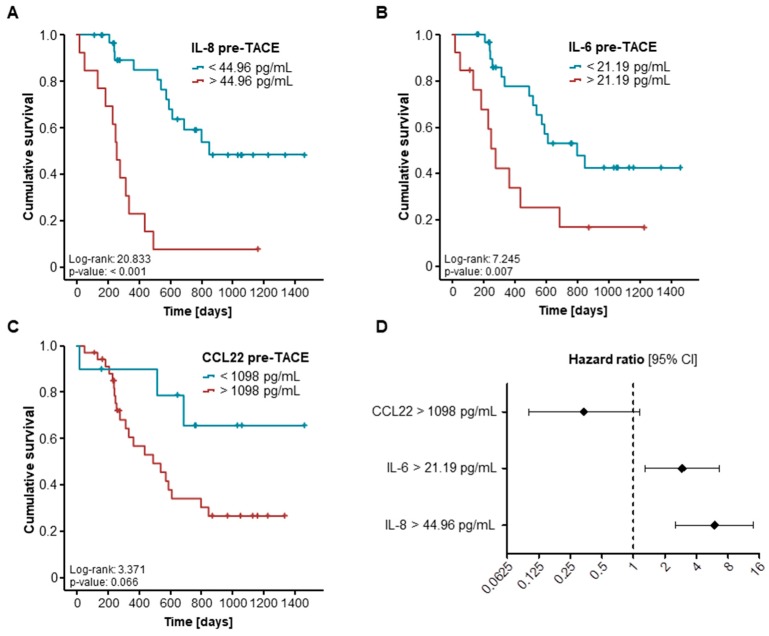
High pre-interventional serum levels of IL-6 and IL-8 are associated with an unfavorable prognosis after TACE. (**A**) Patients with IL-8 serum levels above our defined ideal cut-off value show a significantly impaired long-term survival; (**B**) Pre-interventional IL-6 serum levels discriminate between patients with good and poor prognosis; (**C**) Serum CCL22 levels are unsuitable for the prediction of long-term survival; (**D**) Univariate Cox regression analysis reveals circulating IL-6 and IL-8 but not CCL22 levels as prognostic factors (black diamond: odds ratio, error bars: 95% confidence interval (CI).

Based on the promising findings on the predictive value of IL-8 and IL-6 for an objective response after TACE therapy, we next evaluated a potential influence of initial cytokine serum levels on the patients’ overall survival (OS). We therefore subdivided our cohort of patients into two subgroups with either high (above the 50th percentile) or low (below the 50th percentile) serum levels of the respective cytokine. Here, patients with high pre-interventional IL-8 serum levels (above the 50th percentile of 34.73 pg/mL) had a significantly impaired long-term survival after TACE therapy compared to patients with low IL-8 serum levels (Supplementary Figure S1A). However, serum levels of IL-6 and CCL22 above or below the respective cut-off values were unsuitable for the identification of patients with an unfavorable prognosis (Supplementary Figure S1B,C). We subsequently established ideal prognostic cut-off values by fitting Cox proportional hazard models to the survival status and the survival time and testing for the respective cytokine serum level with the most significant log-rank test as recently described [18]. Using these ideal cut-off values, Kaplan–Meier curve analyses showed a significantly impaired long-term survival for patients with pre-interventional serum IL-8 and IL-6 levels above the respective ideal cut-off values (Figure 3A,B). Patients with IL-8 levels above versus below 44.96 pg/mL showed a median OS of 256 versus 848 days, respectively. Similarly, patients with IL-6 serum levels of 21.19 pg/mL and above showed a significantly reduced median OS compared to those with IL-6 levels below this value (277 versus 799 days). In contrast, the ideal cut-off for CCL22 (1098 pg/mL) was still unable to identify patients with a poor post-interventional prognosis (Figure 3C).

To further substantiate the prognostic relevance of IL-8 and IL-6 in the context of TACE, we next performed univariate Cox regression analysis regarding the OS. Here, pre-interventional IL-8 and IL-6 but not CCL22 serum levels above our defined cut-off values turned out to be significant prognostic factors for the OS (Figure 3D). The hazard ratios (HR) for IL-8 and IL-6 in this setting were 5.977 (*p* < 0.001; 95% CI: 2.548–14.023) and 2.919 (*p =* 0.010, 95% CI: 1.290–6.605), respectively (Table 2). In a second step, we included parameters with a *p*-value of <0.250 in univariate Cox regression analysis (creatinine, CRP, GGT, IL-6 and IL-8) in multivariate testing. Importantly, the prognostic value of both IL-8 and IL-6 serum levels was independent of the patients’ renal function (creatinine), systemic inflammation (CRP), and liver parameters (GGT, Table 2).

### 2.4. Post-Interventional Serum Levels of CCL22, IL-8, and IL-6 Are Unsuitable for the Prediction of TACE Response for 41 and 16 Patients

Out of 54 patients, serum samples at day 1 (41 patients) and day 2 (16 patients) after TACE therapy were available. We subsequently evaluated if post-interventional cytokine serum levels might also have a predictive value for an objective response to TACE. However, when we compared serum levels of IL-8, IL-6, and CCL22 both at day 1 and 2 after TACE in patients who showed an objective response to TACE and non-OR patients, no significant differences became apparent (Supplementary Figure S3). In line with this, post-interventional serum levels of CCL22, IL-6, and IL-8 were unable to predict the response to TACE therapy in a binary logistic regression model.

**Figure 4:**
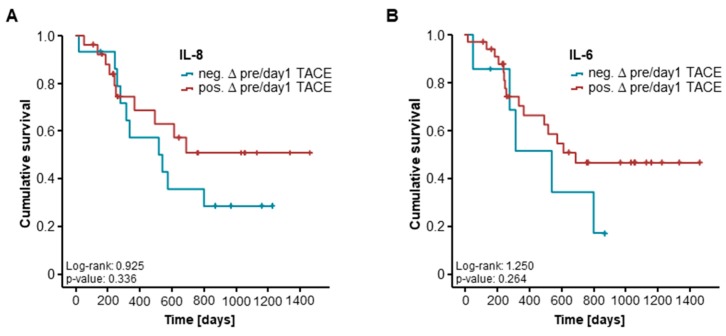

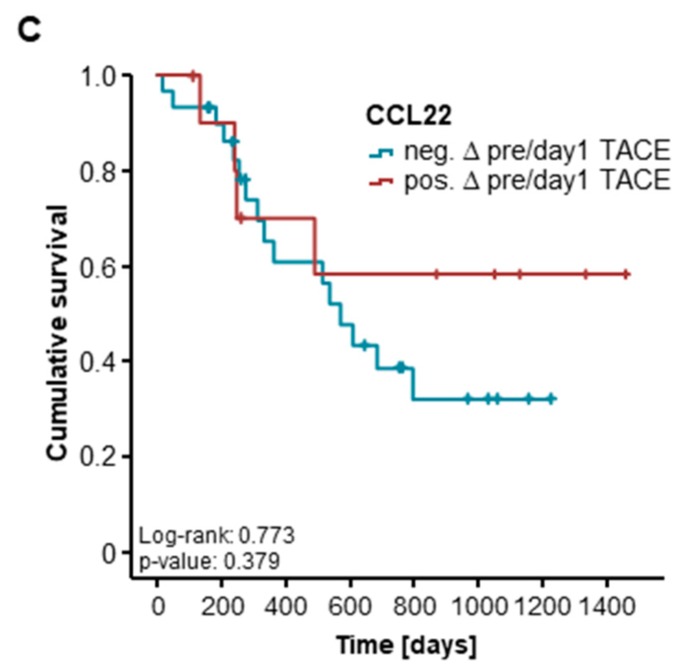
Longitudinal changes of cytokine serum levels and patients’ survival. (**A**) Longitudinal changes of IL-8 serum levels before and at day 1 after TACE are not associated with the patient’s survival after TACE therapy; (**B**,**C**) Patients who showed increasing levels of IL-6 and CCL22 at day 1 after TACE have a similar long-term survival compared to patients with decreasing post-interventional serum levels.

Finally, we assessed if longitudinal changes of cytokine serum levels before and after TACE therapy reflected the patients’ prognosis in this setting. We therefore compared the OS between patients with increasing levels of the respective cytokine at day 1 after TACE and those patients who showed decreasing cytokine levels. Again, Kaplan–Meier curve analysis revealed no significant difference for IL-8, IL-6, and CCL22 for this setting (Figure 4A–C).

## 3. Discussion

Therapeutic concepts for primary and secondary liver cancer have decisively improved over the last decades [2,6]. Not only advances in systemic chemotherapy but also new multimodal therapeutic approaches including surgical resection as well as locally ablative techniques have led to a constantly improving outcome for patients with liver cancer [6,19]. As such, transarterial chemoembolization (TACE) has evolved as the most commonly used primary therapy for HCC, not only for BCLC stage B patients but also for earlier as well as more advanced disease stages [20]. In general, the individual decision at which exact point of a multimodal therapy a patient receives TACE varies significantly between centers, which is not only due to local preferences but also due to a lack of a well-established pre-interventional stratification tool. At present, the decision whether or not a patient with liver cancer should receive TACE therapy is mainly based on exclusion from a curatively intended surgical approach, local tumor expansion (e.g., vascular invasion), liver function, and clinical scores such as the hepatoma arterial-embolisation prognostic (HAP) score [21], while aspects of the tumor biology are less frequently considered.

Here, we show that pre-interventional serum levels of IL-6 and IL-8 not only predict patients’ local tumor response after TACE but are also indicative of the patients’ overall survival (OS). As such, pre-interventional serum levels below our ideal cut-off value of 13.51 pg/mL (IL-6) and 36.36 pg/mL (IL-8) were predictors of an objective response after TACE therapy, independent of the target lesions’ size, and the patients’ liver function (bilirubin) and age (see Figure 2). Moreover, patients with high IL-6 and IL-8 serum levels (above our ideal prognostic cut-off value) showed a significantly reduced OS of 277 (IL-6) and 256 (IL-8) days compared to 799 and 848 days for patients with initial serum levels below these cut-off values (see Figure 3). Again, multivariate Cox regression analysis revealed that this prognostic value was independent of the target lesions’ size or the patients’ liver function and age.

IL-6 and IL-8 both represent immunomodulatory cytokines, which have previously been described as pro-malignant mediators in different tumor entities [14,22]. In HCC, IL-6 promotes multiple stages of tumor development including initial hepatocyte proliferation, the transformation of hepatocytes into HCC progenitor cells (HcPCs), and the progression to HCC nodules and metastases [22]. Binding of IL-6 to its receptor gp80 leads to an intracellular dimerization of its co-receptor gp130, which mainly promotes signal transducer and activator of transcription (STAT) 3 activation via janus kinase (JAK) 1/2 signaling that results in the activation of different pro-malignant downstream pathways [23]. In vitro, IL-6- as well as gp130-deficient mice show a lower HCC incidence and a prolonged survival in the Diethylnitrosamine (DEN) and high-fat diet (HFD) HCC mouse models, which was associated with a reduced expression of STAT3 [22,24]. Interestingly, especially at a later stage of hepatocarcinogenesis, HcPCs acquire an autocrine IL-6 loop due to an intracellular degradation of miRNA Let-7, leading to aggravated cancer progression [25]. In line with this, high IL-6 tumor expression was associated with tumor progression and reduced survival in HCC patients [26]. Thus, elevated serum levels of IL-6 in our cohort of patients might reflect a subgroup of patients with a more aggressive hepatic tumor or tumor microenvironment that is less likely to respond to TACE therapy due to increased tumor regeneration capabilities. This hypothesis might further be corroborated by recently described molecular subtypes of HCC, showing wide variations in IL-6/JAK/STAT activation [27].

Similarly, IL-8 has previously been associated with angiogenesis and cell proliferation, invasion, and migration, all representing important hallmarks of cancer [14]. Serum levels of IL-8 were shown to correlate with tumor size and stage in resectable HCC patients [28]. On a molecular level, IL-8 mediates its biological effects through two highly related seven-transmembrane chemokine receptors—CXC chemokine receptor (CXCR)1 and CXCR2—leading to an activation of the v-Akt Murine Thymoma Viral Oncogene (Akt) and mitogen-activated protein kinase (MAPK) signaling cascade [29]. In vitro, IL-8 directly regulates angiogenesis by enhancing endothelial cell survival and proliferation, and secretion of IL-8 by tumor cells enhances angiogenesis and cell proliferation through an autocrine activation [30]. Interestingly, IL-8/CXCR2-mediated autocrine activation was described to activate intrinsic strategies of malignant tumor cells to evade stress-induced apoptosis [30], and might therefore be one of the underlying mechanisms that leads to TACE refractoriness in the subgroup of patients with high serum IL-8 levels in our cohort. However, further experimental studies, ideally including rodent TACE models [31] are warranted to fully elucidate the molecular mechanisms leading to a poor TACE response in patients with high IL-6 and IL-8 serum levels.

Our study was limited by several points. First, the study was conducted in a small cohort of patients. Second, we included patients with primary and secondary liver cancer. While this latter aspect could limit the entity-specific clinical and pathophysiological conclusion for, e.g., HCC-specific regulatory effects of IL-6 and IL-8, it is interesting to note that both markers had a prognostic effect even in a smaller and heterogeneous cohort. The fact that IL-6 and IL-8 represent rather unspecific inflammatory cytokines, which most likely reflect high tumor regeneration and proliferation capacity, could imply that these markers are rather method specific for TACE therapy and less tumor specific. Thus, while available scoring systems for TACE such as the HAP score [21] are based on HCC-specific parameters like alpha-fetoprotein (AFP), pre-interventional measurements of IL-6 and IL-8 might be a valuable addition to future tumor-entity-independent stratification algorithms for TACE, which could further improve the clinical applicability of these scores. However, this hypothesis would warrant larger clinical studies to fully unravel the prognostic function of IL-6 and IL-8 in the context of TACE and to establish clinically implementable cut-off values that we hope to stimulate with this study.

## 4. Patients and Methods

### 4.1. Study Design and Patient Characteristics

We designed this prospective observational cohort study to evaluate serum levels of IL-6, IL-8, and CCL22 as biomarkers in patients undergoing TACE for primary and secondary hepatic malignancies. A total of 54 patients who were admitted to the Department of Medicine III and who received TACE therapy at the Department of Diagnostic and Interventional Radiology at University Hospital RWTH Aachen were prospectively recruited between 2013 and 2017 and enrolled into this study (detailed patient characteristics are given in Table 3). Serum samples were collected prior to TACE therapy and at days 1 and 2 after the procedure. As a control population, we analyzed 50 healthy, cancer-free blood donors with normal values for blood counts, C-reactive protein, and liver function. The study protocol was approved by the ethics committee of the University Hospital Aachen, RWTH Aachen University, Aachen, Germany (EK 206/09, 05/01/2010) and conducted in accordance with the ethical standards laid down in the Declaration of Helsinki. Written informed consent was obtained from the patients.

### 4.2. Measurement of Serum Cytokine Level

Serum concentrations of IL-6, IL-8, and CCL22 were analyzed by multiplex immunoassay according to the manufacture’s instruction using a Bio-Plex 200 system and Bio-Plex Manager 6.0 software (Bio-Plex Pro Human Chemokine Panel, #171AK99MR2, Bio Rad, Hercules, CA, USA).

### 4.3. Transarterial Chemoembolization (TACE)

All tumors were treated using an emulsion of a chemotherapeutic agent and an embolic agent diluted with iodized contrast (Ultravist 300, Bayer Vital GmbH, Leverkusen, Germany). HCCs were treated with Doxorubicine and ethiodized oil (Lipiodol, Guerbet LLC, Bloomington, IN, USA). Other tumor entities, such as colorectal, gastric, or pancreatic cancer, were treated using a chemotherapeutic agent in accordance with the specific guidelines and Lipiodol, degradable starch microspheres (EmboCept S, PharmaCept GmbH, Berlin, Germany), or drug-eluting beads (DcBeads, BTG International Ltd., London, UK). All chemoembolization procedures were conducted via the right femoral artery. Hepatography as well as a contrast-enhanced cone-beam CT in late arterial contrast phase were performed using a 2.4F- or 2.7F-microcatheter. Depending on the tumor type and the number, size, localization, and arterial supply of the tumor, a superselective (subsegmental), selective (segmental), or nonselective (lobar) approach was performed.

### 4.4. Evaluation of TACE Response

All patients underwent either a multidetector CT with multiphasic, contrast-enhanced acquisitions in native, arterial, portal venous, and late-venous phase or a multiphasic, contrast-enhanced liver MRI (1,5T, Philips Medical Systems DMC GmbH, Hamburg, Germany) not earlier than 4 weeks prior and at approximately four weeks after TACE. The median time to the follow-up imaging after TACE was 34 days. All CT and MRI scans were analyzed according to response evaluation criteria in solid tumors (RECIST) 1.1 criteria for non-arterially enhanced tumor entities [32] and mRECIST criteria for HCC [33]. Overall tumor response was classified using the standard nomenclature for RECIST 1.1 and mRECIST: complete response (CR), partial response (PR), stable disease (SD), and progressive disease (PD). Complete response and partial response were further summarized into objective response (OR) [17].

### 4.5. Statistical Analysis

Statistical analyses were performed as recently described [34].Data are given as median and range to reflect the skewed distribution of analysis on human samples. Nonparametric data were compared using the Mann–Whitney *U* test and, for multiple comparisons, the Kruskal–Wallis test. Box plot graphics display a statistical summary of the median, quartiles, and ranges. ROC curves were generated by plotting sensitivity against 1-specificity. The optimal cut-off values for ROC curves were established using the Youden index. The predictive value of serum cytokine levels with respect to an objective response to TACE therapy was tested using a binary logistic regression model. The odds ratio and the 95% confidence interval are displayed. Kaplan–Meier curves were plotted to display the impact on survival. The log-rank test was used to test for differences between subgroups in Kaplan–Meier curve analysis. The optimal cut-off value for the identification of patients with an impaired OS was established using recently published biometric software, which fits Cox proportional hazard models to the dichotomized survival status (dead versus alive) and the survival variable (survival time). The optimal cut-off is defined as the point with the most significant (log-rank test) split. [18]. The prognostic value of variables was further tested by univariate and multivariate analysis in the Cox regression model. The hazard ratio (HR) and the 95% confidence interval are displayed. All statistical analyses were performed with SPSS 23 (SPSS, Chicago, IL, USA). A *p*-value of <0.05 was considered statistically significant (* *p* < 0.05; ** *p* < 0.01; *** *p* < 0.001).

## Figures and Tables

**Table 1:** Serum levels of laboratory markers.

Parameter	TACE Patients Median (Range)	Healthy Controls Median (Range)
Interleukin (IL)-8 pre-TACE (pg/mL)	35.96 (7.69–381.77)	7.78 (0–158.05)
IL-8 day 1 post-TACE (pg/mL)	40.17 (12.48–323.73)	-
IL-8 day 2 post-TACE (pg/mL)	39.76 (12.39–332.86)	-
IL-6 pre-TACE (pg/mL)	12.17 (3.49–187.88)	3.59 (0–17.57)
IL-6 day 1 post-TACE (pg/mL)	31.67 (7.90–164.59)	-
IL-6 day 2 post-TACE (pg/mL)	31.29 (10.34–566.03)	-
CC-chemokine ligand (CCL) 22 pre-TACE (pg/mL)	835.12 (101.57–1872.29)	934.48 (101.57–1925.91)
CCL22 day 1 post-TACE (pg/mL)	780.66 (110.19–1516.91)	-
CCL22 day 2 post-TACE (pg/mL)	644.16 (203.83–1167.72)	-
Bilirubin (mg/dL)	0.73 (0.26–2.43)	0.41 (0.1–1.46)

**Table 2:** Univariate and multivariate Cox regression analysis for the prediction of overall survival.

Parameter	Univariate Cox Regression		Multivariate Cox Regression	
	*p*-Value	Hazard Ratio (95% CI)	*p*-Value	Hazard Ratio (95% CI)
IL-6 > 21.19 pg/mL	0.010	2.919 (1.290–6.605)	0.009	3.446 (1.355–8.768)
IL-8 > 44.96 pg/mL	<0.001	5.977 (2.548–14.023)	0.009	4.679 (1.471–14.889)
Size of target lesion	0.277	1.006 (0.995–1.017)		
Age	0.444	1.014 (0.978–1.052)		
Sex	0.898	1.062 (0.424–2.662)		
Creatinine	0.238	1.426 (0.791–2.573)	0.181	1.515 (0.824–2.787)
Potassium	0.325	1.420 (0.706–2.858)		
Leucocytes	0.270	1.102 (0.928–1.308)		
Alanine transaminase (ALT)	0.807	0.999 (0.992–1.006)		
Lactate dehydrogenase (LDH)	0.943	1.000 (0.997–1.003)		
Bilirubin	0.857	1.075 (0.490–2.357)		
γ-Glutamyl transpeptidase (GGT)	0.043	1.001 (1.000–1.002)	0.654	1.000 (0.999–1.002)
C-reactive protein (CRP)	0.019	1.021 (1.003–1.040)	0.487	0.991 (0.965–1.017)

**Table 3:** Characteristics of study population.

Patient Characteristics	Study Cohort
TACE patients	54
Sex (%):	
male–female	79.6–20.4
Age (years, median and range)	66.5 (37–89)
Hepatic malignancy (%):	
HCC	81.5
Liver metastasis (CRC)	9.3
Liver metastasis (gastric cancer)	1.8
Liver metastasis (pancreatic)	3.7
Liver metastasis (CCA)	3.7
Size of target lesion (mm, median and range)	29 (10–129)
OR to TACE therapy (%):	
Yes–No	43.2–56.8
Deceased during follow-up (%):	
Yes–No	56.6–43.4

TACE, transarterial chemoembolization; HCC, hepatocellular carcinoma; CRC, colorectal carcinoma; CCA, cholangiocarcinoma; OR, objective response.

## Supplementary Materials

The following are available online at http://www.mdpi.com/1422-0067/19/6/1766/s1.

## Abbreviations

AFP	Alpha-fetoprotein
Akt	v-Akt Murine Thymoma Viral Oncogene
ALT	alanine transaminase
ART	Assessment for Retreatment with TACE
AUC	area under the curve
BCLC	Barcelona Clinic Liver Cancer
CCL	CC-chemokine ligand
CRC	colorectal carcinoma
CRP	C-reactive protein
CXCR	CXC chemokine receptors
DEN	Diethylnitrosamine
ECLIA	electrochemiluminescence immunoassay
GGT	γ-Glutamyl transpeptidase
HAP	Hepatoma arterial-embolisation prognostic
HCC	hepatocellular carcinoma
HcPCs	HCC progenitor cells
HFD	high-fat diet
HR	hazard ratio
IL	interleukin
JAK	janus kinase
LDH	Lactate dehydrogenase
MAPK	mitogen-activated protein kinase
OR	objective response
OS	overall survival
RECIST	Response Evaluation Criteria in Solid Tumors
RFA	radiofrequency ablation
ROC	receiver operator characteristics
SNACOR	tumour Size and Number, baseline Alpha-fetoprotein, Child-Pugh and Objective radiological Response
STAT3	signal transducer and activator of transcription 3
TACE	transarterial chemoembolization
