# Supplementary material for: IL-6 and IL-8 Serum Levels Predict Tumor Response and Overall Survival after TACE for Primary and Secondary Hepatic Malignancies
Source: Int J Mol Sci. 2018 Jun 14;19(6):1766. doi: 10.3390/ijms19061766 (PMC6032291; doi:10.3390/ijms19061766)
Supplement: Supplementary file 1 [file ijms-19-01766-s001.zip › ijms-306140 supplementary/Figures' caption.pdf]

Supplementary Figure 1. (A) Serum levels of IL-8 before TACE significantly correlate with the target lesions' longitudinal changes of size after TACE. (B and C) Serum concentrations of IL-6 and CCL22 do not significantly correlate with a change of size of the target lesion after TACE

Supplementary Figure 2. (A) Patients with high pre-interventional IL-8 serum levels (above the 50th percentile) have a significantly impaired long term survival after TACE therapy compared to patients with low IL-8 serum levels. (B and C) Serum levels of IL-6 and CCL22 above and below the 50th percentile are unable to identify patients with an unfavourable prognosis.

Supplementary Figure 3. (A-C) Serum levels of IL-8, IL-6 and CCL22 at day 1 after TACE are unaltered between patients who showed an objective response (OR) to TACE and non-responding (non-OR) patients. (D-F) Serum levels of IL-8, IL-6 and CCL22 at day 2 after TACE do not differ between OR and non-OR patients.
