# Supplementary figures and images for: IL-6 and IL-8 Serum Levels Predict Tumor Response and Overall Survival after TACE for Primary and Secondary Hepatic Malignancies
Source: Int J Mol Sci. 2018 Jun 14;19(6):1766. doi: 10.3390/ijms19061766 (PMC6032291; doi:10.3390/ijms19061766)

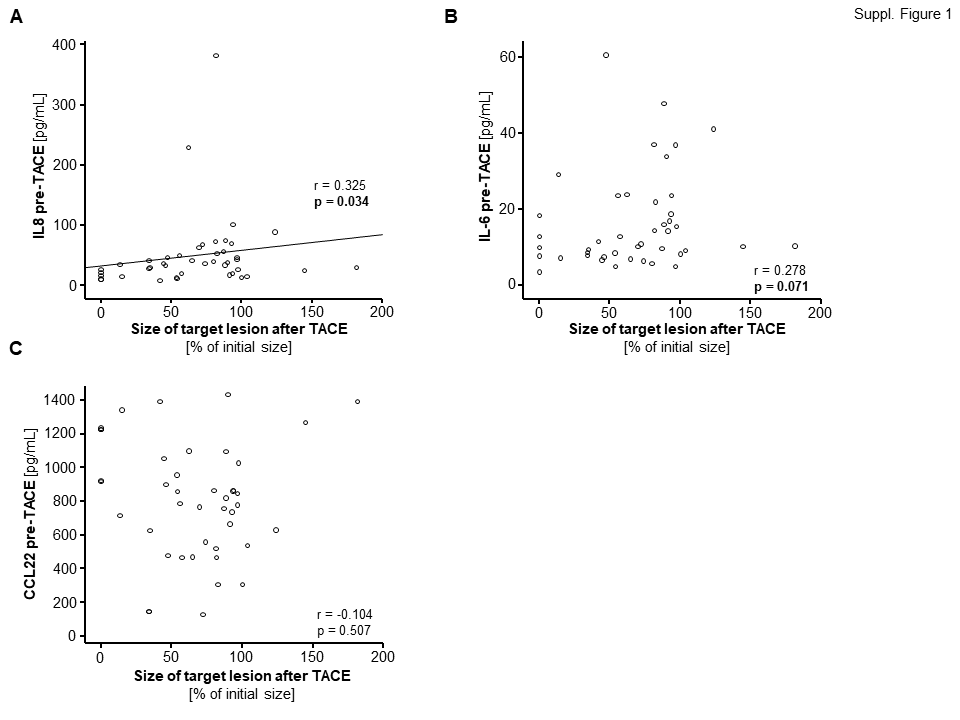

Supplement: Supplementary file 1 [file ijms-19-01766-s001.zip › ijms-306140 supplementary/Supplementary Figure 1.TIF]

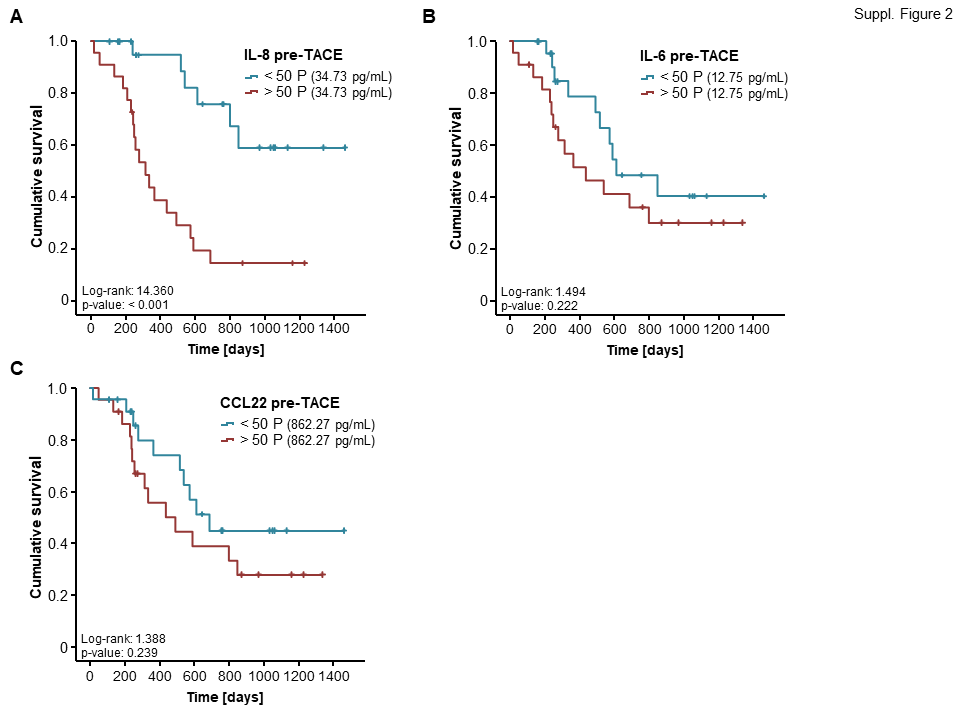

Supplement: Supplementary file 1 [file ijms-19-01766-s001.zip › ijms-306140 supplementary/Supplementary Figure 2.TIF]

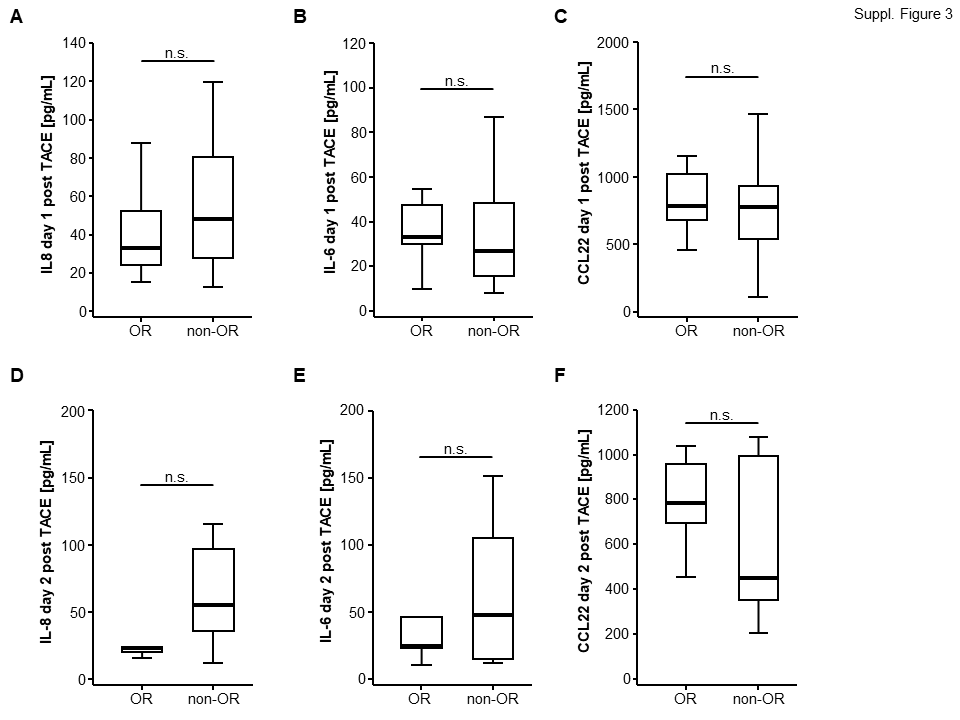

Supplement: Supplementary file 1 [file ijms-19-01766-s001.zip › ijms-306140 supplementary/Supplementary Figure 3.TIF]
